# Interpreting the Molecular Mechanisms of Yinchenhao Decoction on Hepatocellular Carcinoma through Absorbed Components Based on Network Pharmacology

**DOI:** 10.1155/2021/6616908

**Published:** 2021-05-19

**Authors:** Jijia Sun, Tao Han, Tao Yang, Yunhui Chen, Jihan Huang

**Affiliations:** ^1^Center for Drug Clinical Research, Shanghai Traditional Chinese Medicine Health Service Collaborative Innovation Center, Shanghai University of Traditional Chinese Medicine, Shanghai 201203, China; ^2^Department of Cardiology, Cardiovascular Research Institute, Shuguang Hospital Affiliated to Shanghai University of Traditional Chinese Medicine, Shanghai 201203, China; ^3^College of Basic Medicine, Chengdu University of Traditional Chinese Medicine, Chengdu 610075, China

## Abstract

To investigate the mechanisms through which Yinchenhao decoction (YCHD) inhibits hepatocellular carcinoma (HCC), we analyzed YCHD ingredients absorbed into the bloodstream by using network pharmacology. We conducted a weighted gene coexpression network analysis on gene expression data collected from the Gene Expression Omnibus and The Cancer Genome Atlas databases to derive an HCC gene set; moreover, we used four online prediction system databases to predict the potential targets of YCHD ingredients absorbed into the bloodstream. We discovered that YCHD directly interfered with 17 HCC-related disease targets. Subsequent gene ontology enrichment analyses of these 17 disease targets revealed that YCHD exhibited effects through 17 biological processes, 7 molecular functions, and 9 cellular components. Kyoto Encyclopedia of Genes and Genomes (KEGG) enrichment analyses indicated 14 pathways through which YCHD inhibits HCC. We observed similar trends in how the 17 small molecules interfered with the key target set. We surmised that YCHD inhibits HCC by regulating inflammatory and metabolic pathways. Network pharmacological analysis of YCHD ingredients absorbed into the bloodstream may provide new insights and serve as a new method for discovering the molecular mechanisms through which YCHD inhibits HCC.

## 1. Introduction

Hepatocellular carcinoma (HCC) is the fourth most common cancer and the third most deadly cancer in China, accounting for 85%–90% of primary malignant liver tumors [1]. Although the multikinase inhibitor *sorafenib* has been approved as a first-line treatment for late-stage HCC because of its favorable antiangiogenesis effects, it increases patients' survival time by only a few months [2]. Traditional Chinese medicine (TCM), which has a long history of clinical application, exhibits multimolecule, multitarget, and synergistic effects. TCM has subsequently become a valuable alternative for HCC treatment. Therefore, the effectiveness of TCM in inhibiting HCC merits further investigation [3].

Introduced in *Shanghan Lun* (*Treatise on Cold Damage Diseases*), Yinchenhao decoction (YCHD)—which consists of *yinchen* (Artemisiae Scopariae Herba), *dahuang* (Radix et Rhizoma Rhei), and *zhizi* (Gardeniae Fructus)—is a popular remedy in TCM and is commonly used to treat *yang* jaundice. A study identified 160 potential targets related to 16 diseases, including cancer; of these targets, 96 were related to cancer [4]. Concerning oral TCM compounds (i.e., medicine comprising two or more ingredients), the ingredients must undergo absorption, distribution, metabolism, and excretion before they enter the bloodstream; only molecules that complete this process are considered active. These molecules merge with targets in the organism and then exhibit medicinal effects. In a preliminary study, we obtained active chemical molecules by using oral bioavailability (OB) screening [5], drug-likeness (DL) assessments [6], and intestinal epithelial permeability (Caco-2) screening. In that study, we were able to predict which molecules were beneficial; however, their effects were minimal if the molecule content was low or if the molecules were metabolized quickly. Sun et al. used the highly sensitive ultraperformance liquid chromatography–tandem mass technology to identify the 69 compounds in YCHD, among which 41 were absorbed into the bloodstream [7]. Wu et al. conducted a study on major anti-HCC components in YCHD in vitro and in vivo [8]. Nevertheless, the active molecules in YCHD compounds and the mechanisms through which these compounds mitigate HCC remain relatively unknown.

TCM incorporates complex chemical compositions, and effective research methods for examining these compositions are limited; additionally, the pharmacological mechanisms governing TCM have yet to be systematically explained [9]. Systems pharmacology [10] is an emerging discipline that involves systematically studying the interaction between medicine and organisms as well as the patterns, effects, and mechanisms of such medicine. Network pharmacology [11] is used to study active substances in TCM, identify the targets affected by these substances, determine the relationships between effective substances and diseases, and elucidate the molecular mechanisms governing the activities of TCM compounds. Therefore, systems pharmacology- and network pharmacology-based studies offer new perspectives and insights into TCM compounds. Regarding the use of network pharmacology to study TCM, our laboratory has performed numerous experiments and explored the pharmacological mechanisms governing *zuojin* pills [12], *huanglian jiedu* decoction [13], and *huachansu* capsules [14] in the treatment of HCC.

**Figure 1:**
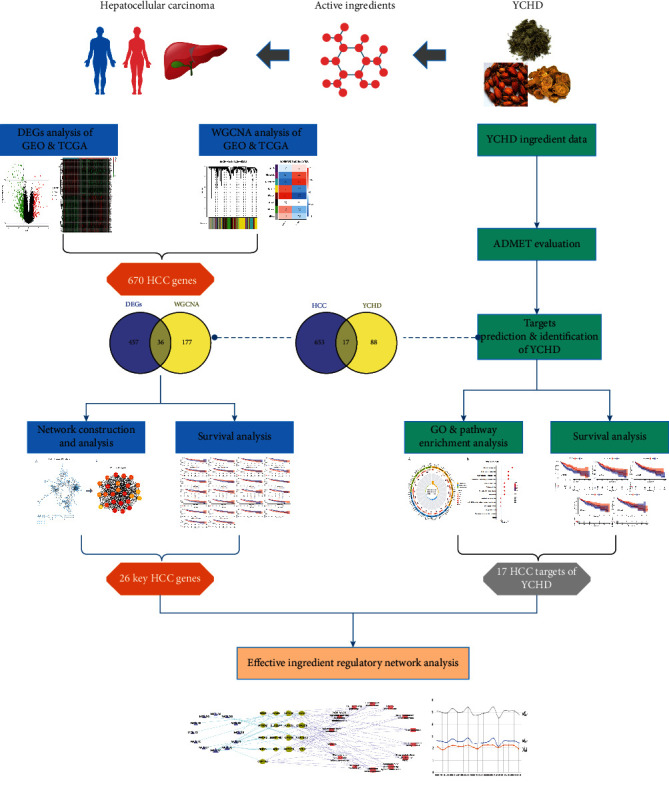
Network pharmacology-based flowchart depicting how YCHD inhibits HCC.

Accordingly, the present study analyzed data obtained from the Gene Expression Omnibus (GEO) and The Cancer Genome Atlas (TCGA) databases to identify the differentially expressed genes (DEGs) of HCC. On the basis of the YCHD ingredients absorbed into the bloodstream, network pharmacology was then used to identify active HCC-inhibitive pharmaceutical ingredients and their possible molecular targets. The workflow of our bioinformatics and network pharmacology analyses of the effects of YCHD on HCC is presented in Figure 1. The results may serve as a basis for subsequent experimental research.

## 2. Materials and Methods

### 2.1. Data Collection

The GEO is a public gene expression profile database of the National Center for Biotechnology Information, National Institutes of Health (USA). By mining public databases, medical researchers can obtain a clearer understanding of the molecular mechanisms underlying the onset of cancer. Such an understanding can enhance early detection, diagnosis, and treatment of various cancers [15]. In the current study, we used “liver cancer” and “hepatocellular carcinoma” as search terms in the high-throughput GEO database and collected disease-related gene expression profile chips. After analyzing and comparing the different chips, we selected the GSE121248 chip for analysis. This chip originated from the GPL570 [HG-U133_Plus_2] Affymetrix Human Genome U133 Plus 2.0 Array platform, which contains 70 liver cancer samples and 37 normal samples. TCGA is a joint project initiated by the American National Cancer Institute and the National Human Genome Research Institute in 2006. TCGA is the largest cancer gene database worldwide and contains a wealth of clinical information [16]. We searched TCGA for (and subsequently downloaded) liver cancer-related RNA-seq gene expression data and the corresponding clinical data files, which contained 373 tumor samples and 49 normal samples.

### 2.2. DEG-Based Disease Gene Analysis

We used the limma package (version: 3.42.2) in R language to analyze DEGs identified in the GSE121248 and TCGA data. Subsequently, we filtered out upregulated and downregulated DEGs from the GEO and TCGA chip data by using the following conditions:|log_2_FC| > 1.0 and adj.*p*.value < 0.05. Finally, we employed the ggplot2 package (version: 3.3.1) and pheatmap package (version: 1.0.12) to draw volcano maps and heat maps corresponding to the DEGs, and we used Venny 2.1 (https://bioinfogp.cnb.csic.es/tools/venny/index.html) to determine the intersection of TCGA and GEO data in order to derive the differential gene analysis-based gene set *G*_*D*_.

### 2.3. Weighted Gene Coexpression Network Disease Gene Analysis

We performed a weighted gene coexpression network analysis (WGCNA) to analyze the gene expression data from the GEO and TCGA. From this analysis, we identified modules and hub genes related to clinical phenotypes (i.e., normal and disease-based).

A gene coexpression network was constructed using a weighted gene expression correlation network analysis. The soft-threshold power was extracted from PowerEstimated and the topological overlap matrix (TOM). Subsequently, on the basis of the weighted gene expression correlation network, the TOM was used to cluster all the genes, and dynamic tree cutting was employed to identify various gene modules. To determine the correlation between the different modules of the clustered genes, a minimum gene module size of 50 and a dynamic shear height value of 0.25 were set. The different gene modules are represented by various colors. Finally, the correlation between different gene modules and clinical phenotypes was calculated, and a heat map of the correlation between the modules and clinical phenotypic traits was drawn. According to the correlation between the gene module and the normal and control groups, we determined modules with the highest positive and negative correlation values in the GEO and TCGA data, respectively. Subsequently, gene modules with the highest positive and negative correlation values in the GEO and TCGA data were intersected, and two intersecting genes were combined to constitute the HCC gene set *G_W_* through the WGCNA.

### 2.4. HCC Protein–Protein Interaction Network Construction and Analysis

We combined the HCC gene set *G*_*D*_ obtained from the DEG-based analyses with the HCC gene set *G*_*W*_ obtained from the WGCNA to form the liver cancer disease gene set *G*_HCC_; these gene sets were imported into the STRING database (version: 11.0; https://string-db.org/). Regarding the parameter settings, we set the parameter “organism” to “homo sapiens” and set the “combine score” threshold to 0.9 to produce an interaction network of all gene targets. We then used Cytoscape (version: 3.7.2, https://cytoscape.org/) to produce a protein–protein interaction (PPI) network for the target proteins.

Furthermore, we used a Cytoscape-based CytoHubba plug-in to mine the core genes in the PPI network and selected the maximal clique centrality (MCC) algorithm for the network. We also used default values for the other parameters and calculated the core gene set *G*_*P*_ in the liver cancer PPI network.

### 2.5. Gene Ontology Function and Kyoto Encyclopedia of Genes and Genomes Pathway Enrichment Analysis

The Gene Ontology (GO) resource is a standard, structured biological model constructed by the GO Consortium in 2000 and encompasses information on the biological processes, molecular functions, and cellular components of genes. A group of genes, rather than a single gene, typically participates in a biological process or pathway. Enrichment analyses are performed on the premise that if a biological process or pathway is known to be abnormal, genes that function together are extremely likely to be selected into the gene set related to this process or pathway.

We used OmicShare (http://www.omicshare.com/tools) to annotate the GO functions for the HCC gene set *G*_HCC_ and applied the clusterProfiler package (version: 3.14.3) to conduct Kyoto Encyclopedia of Genes and Genomes (KEGG) pathway enrichment analyses. Moreover, we selected genes using the conditions *p*.value < 0.05 and *q*.value < 0.05 and employed Cytoscape to construct an HCC target–pathway network according to the enrichment analysis results.

### 2.6. Discovery of Key Disease Genes through Survival Analysis

We determined the key gene set *G*_*K*_ through a survival analysis and included all genes that satisfied the following conditions in our subsequent analyses: (1) genes in the intersection between the gene sets *G*_*D*_ and *G*_*W*_ obtained from the DEG-based analyses and WGCNA, respectively, and (2) the core gene set *G*_*P*_ obtained from the PPI network analyses described in Section 2.4.

We downloaded clinical data files from TCGA. After organizing the data, we used the survival package (version: 3.1) and survminer package (version: 0.4.7) to conduct survival analyses (where *p* < 0.05) and plot gene survival curves.

### 2.7. Collection of Effective YCHD Ingredients and Assessment of Absorption, Distribution, Metabolism, Excretion, and Toxicity

We referenced data on YCHD ingredients collected in animal experiments performed in related studies [7]; we also collected chemical information or data on effective YCHD ingredients from the PubChem database (https://pubchem.ncbi.nlm.nih.gov/) and organized them according to PubChem name and CID, molecular formula, and the simplified molecular-input line-entry system (SMILES) strings (hereafter referred to as canonical SMILES). Additionally, we used Advanced Chemistry Development, Inc. (ACD/Labs) software (version: 2019) and the SwissADME online prediction system (http://www.swissadme.ch/) to evaluate and analyze the absorption, distribution, metabolism, excretion, and toxicity (ADMET) of the ingredients.

### 2.8. Prediction and Identification of Potential Targets of Effective Ingredients

According to the canonical SMILES corresponding to the effective YCHD ingredients, we used four online prediction systems (i.e., HitPick (http://mips.helmholtz-muenchen.de/proj/hitpick), the similarity ensemble approach (SEA) (http://sea.bkslab.org/), SwissTargetPrediction (http://www.swisstargetprediction.ch/), and version 5.0 of the Search Tool for Interacting Chemicals (STITCH) (http://stitch.embl.de/)) to predict and select the potential targets of effective YCHD ingredients. The thresholds were as follows: precision ≥ 80%; max Tc ≥ 0.9; probability ≥ 0.8; and combined score ≥ 0.8. Concurrently, all the predicted ingredients and targets were merged and sorted; the UniProt database (https://www.uniprot.org/) was used to verify target information, and Cytoscape was used to construct an ingredient–effect network for YCHD.

We obtained the intersection between the predicted target gene set and the disease gene set *G*_*W*_, identified the potential targets of YCHD effective in treating HCC, and obtained the intersection gene set *G*_YH_. Additionally, we conducted survival analyses on all genes in the intersection gene set *G*_YH_.

### 2.9. Calculation Model-Based Effective Ingredient Regulatory Network Analysis

In practice, some TCM ingredients do not exhibit direct effects on disease-related targets. However, such ingredients occasionally affect or regulate key disease targets by interfering with neighboring targets. Therefore, we adopted a network calculation model to detect small molecules consistent with the aforementioned description.

We downloaded a high-quality, all-human PPI background network integrating 15 commonly used databases and selected a high-quality PPI incorporating five types of evidence. The PPI network contained a total of 16,677 proteins and 243,603 interactive relationships, and it was subsequently set as the background network of the current study to determine the intensity of YCHD ingredients' interference with and regulation of key genes and the key gene set *G*_*K*_.

#### 2.9.1. Analysis of Key Gene Set Interference with the Target Sets of Different Ingredients

We performed the analyses by using three network topological distances 〈*d*_*AB*_〉 (i.e., 〈*d*_*AB*_^*S*^〉, 〈*d*_*AB*_^*C*^〉, and 〈*d*_*AB*_^*K*^〉). These distances can be expressed as follows:

(1) $$\begin{array}{c} d_{AB}^{S}=\frac{1}{A\times B}\underset{a\in A,\;b\in B}{\sum }d\left(a,b\right),\\ d_{AB}^{C}=\frac{1}{A+B}\left(\underset{a\in A}{\sum }\min_{b\in B}d\left(a,b\right)+\underset{b\in B}{\sum }\min_{a\in A}d\left(a,b\right)\right),\\ d_{AB}^{K}=\frac{-1}{A+B}\left(\underset{a\in A}{\sum }\ln\underset{b\in B}{\sum }\frac{e^{-\left(d\left(a,b\right)+1\right)}}{B}+\underset{b\in B}{\sum }\ln\underset{a\in A}{\sum }\frac{e^{-\left(d\left(a,b\right)+1\right)}}{A}\right), \end{array}$$

where *A* is the target set of an ingredient, ‖*A*‖ is the number of targets in the target set, *B* is the key gene set, ‖*B*‖ is the number of targets in the key gene set, and *d*(*a*, *b*) is the distance between two nodes in the PPI network.

#### 2.9.2. Analysis of Network Proximity between the Key Gene Set and the Target Sets of Different Ingredients

*S*_*AB*_^*P*^ can be expressed as follows:

(2) $$\begin{array}{c} {\text{S}}_{AB}^{P}=\langle d_{AB}^{S}\rangle -\frac{\langle d_{AA}^{S}\rangle +\langle d_{BB}^{S}\rangle }{2}, \end{array}$$

where 〈*d*_*AA*_^*S*^〉 is the average distance between the targets of an ingredient, 〈*d*_*BB*_^*S*^〉 is the average distance between key genes, and 〈*d*_*AB*_^*S*^〉 is the average distance between the target set of an ingredient and the key target set. *S*_*AB*_^*P*^ < 0 signifies that the target set *A* of an ingredient and the key gene set *B* are close together in the network topological structure, indicating that the ingredient can regulate the key gene set *B* by interfering with the target set *A*. *S*_*AB*_^*P*^ ≥ 0 signifies that the target set *A* of an ingredient and the key gene set *B* are far apart in the network topological structure, indicating that the ingredient cannot markedly regulate the key gene set *B*. Therefore, by calculating 〈*d*_*AB*_^*S*^〉, 〈*d*_*AB*_^*C*^〉, 〈*d*_*AB*_^*K*^〉, and *S*_*AB*_^*P*^, one can determine whether YCHD contains ingredients that directly or indirectly affect key disease targets by interfering in certain target (set) proteins, thereby achieving the goal of alleviating or curing diseases.

In this study, we used R language (version 3.6.2) and the igraph package (version 1.2.5) to complete the aforementioned programming, calculations, and analyses.

## 3. Results

### 3.1. DEG-Based Disease Gene Analysis Results

**Figure 2:**
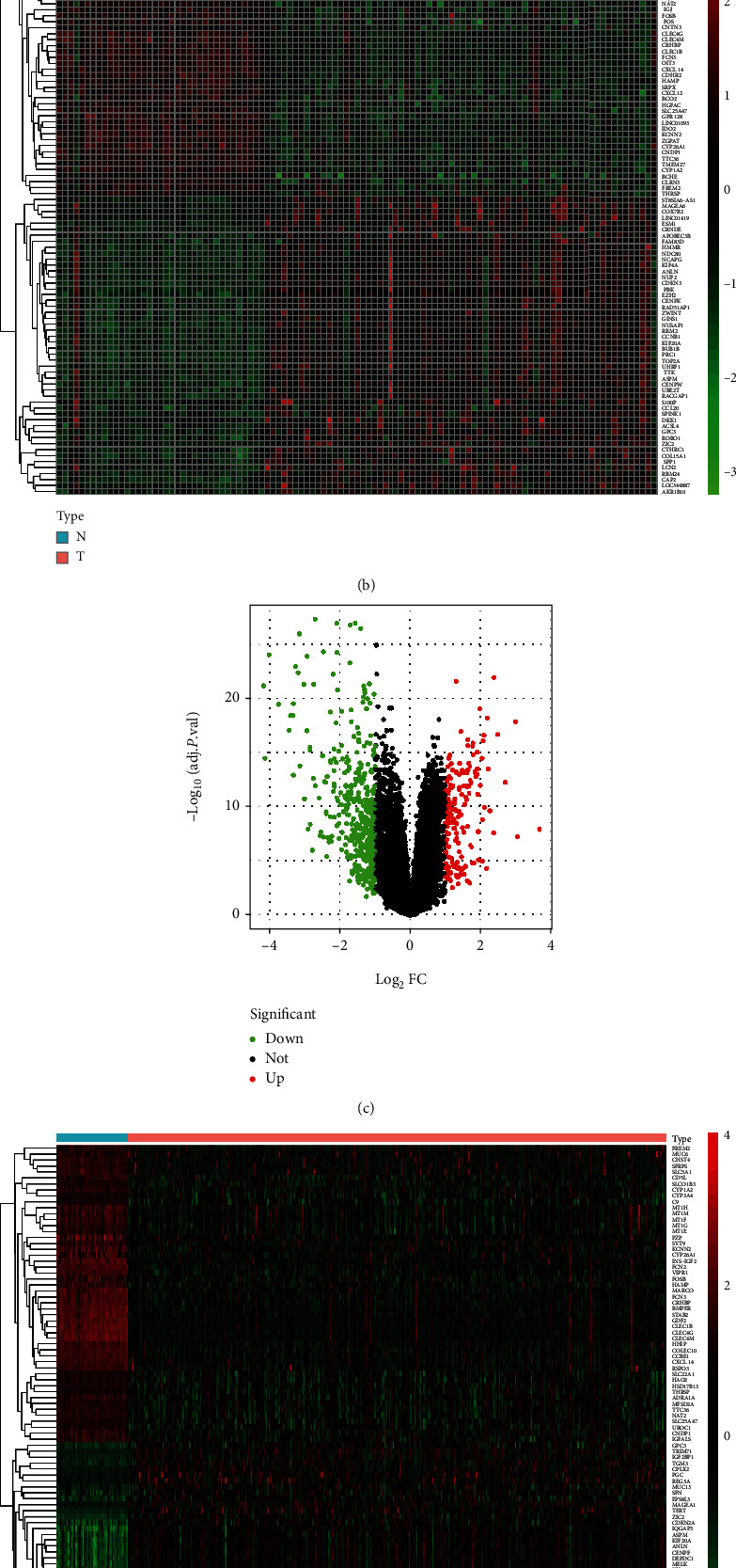
(a) Volcano map of the GEO-based DEG analysis. (b) Heat map of the GEO-based DEG analysis. (c) Volcano map of TCGA-based DEG analysis. (d) Heat map of TCGA-based DEG analysis. (e) Venn diagram of the intersection between the GEO and TCGA DEGs.

We used the limma package to conduct a DEG analysis on liver cancer data obtained from GSE121248 and TCGA. For GSE121248, we obtained 558 DEGs, 167 and 413 of which were upregulated and downregulated genes, respectively. For TCGA, we obtained 2713 DEGs, 1024 and 1689 of which were upregulated and downregulated genes, respectively. Figures 2(a)–2(d) illustrate the volcano and heat maps of the analysis results. By obtaining the intersection of TCGA and GSE121248 data, we identified that the two sets of data shared 493 genes (*G*_*D*_ set) (Figure 2(e)).

### 3.2. WGCNA Results for Disease Genes

**Figure 3:**
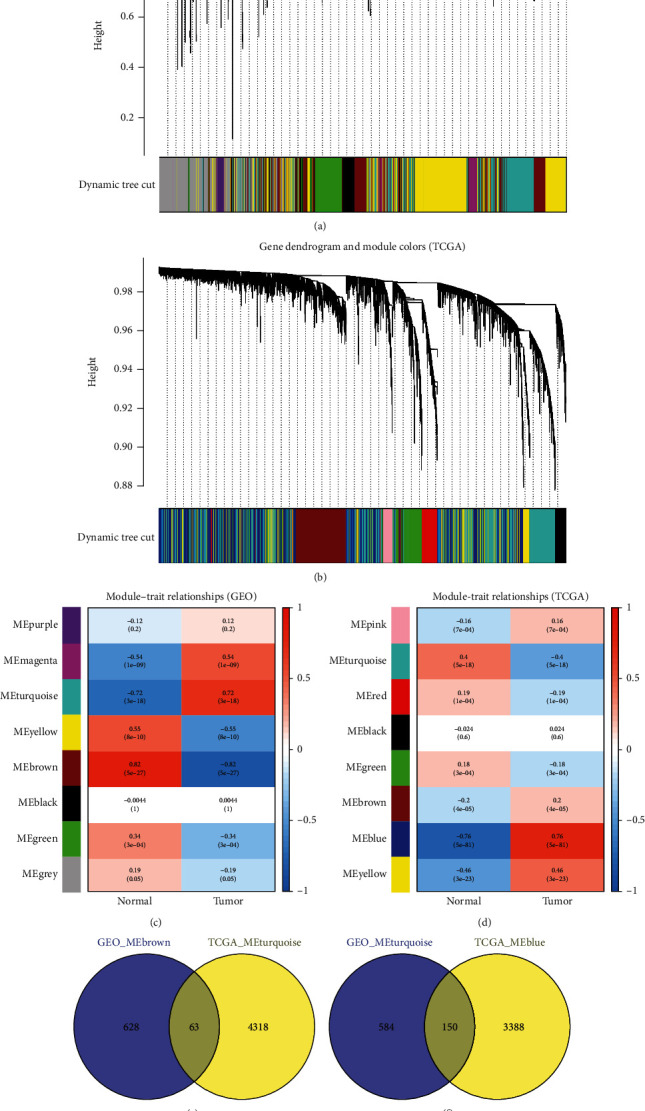
(a) Dynamic tree cutting-based GEO gene module identification results. (b) Correlation between different GEO gene modules and phenotypes. (c) Dynamic tree cutting-based GEO gene module identification results. (d) Correlation between different TCGA gene modules and phenotypes. (e) Intersection between the positively correlated module collected from the GEO-based WGCNA (i.e., MEbrown) and that collected from TCGA-based WGCNA (i.e., MEturquoise). (f) Intersection between the negatively correlated module collected from the GEO-based WGCNA (i.e., MEturquoise) and that collected from TCGA-based WGCNA (i.e., MEblue).

We performed a WGCNA on the gene expression profile data collected from GSE121248 and identified that the modules most positively and negatively correlated with the normal and disease phenotypes were MEbrown (0.82) and MEturquoise (−0.72), which contained 691 and 33 genes, respectively. Conversely, the WGCNA performed on TCGA data revealed that the modules most positively and negatively correlated with the normal and disease phenotypes were MEturquoise (0.4) and MEblue (−0.76), which contained 4381 and 3538 genes, respectively (Figures 3(a)–3(d)).

We obtained the intersection between the positively correlated module collected from the GEO-based WGCNA (i.e., MEbrown) and that collected from TCGA-based WGCNA (i.e., MEturquoise), identifying 63 common genes. Subsequently, we obtained the intersection between the negatively correlated module collected from the GEO-based WGCNA (i.e., MEturquoise) and that collected from TCGA-based WGCNA (i.e., MEblue), identifying 150 common genes. We then combined the two sets of common genes (in total, 213 common genes) to form the WGCNA-based HCC gene set *G*_*W*_ (Figures 3(e) and 3(f)).

### 3.3. HCC PPI Network Analysis Results

**Figure 4:**
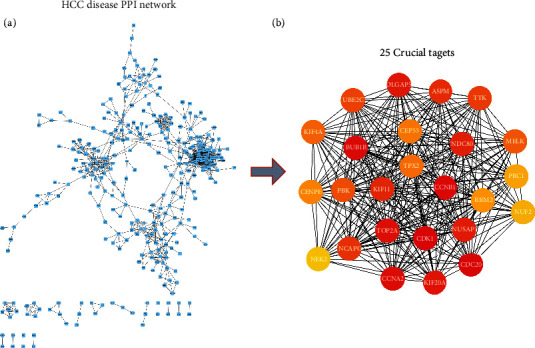
(a) HCC PPI network. (b) The 25 crucial targets in the PPI network obtained using the MCC algorithm. In the figure, the degree value of the node is presented as a gradient from red to green (large to small) according to the degree of the target in the entire PPI network. The darker the color is, the greater the degree value of the node is.

We combined the 493 genes (in gene set *G*_*D*_) obtained from the DEG analysis with the 213 genes (in gene set *G*_*W*_) obtained from the WGCNA to form a liver cancer disease gene set *G*_HCC_, which contained 670 genes. Subsequently, we imported the 670 genes into the STRING database to generate the HCC PPI network. We then applied the MCC algorithm for analysis, identifying 25 genes in the PPI network with much higher scores compared with those of the other genes. The 25 genes were thus determined to be core gene set *G*_*P*_ in the HCC PPI network (Figures 4(a) and 4(b)).

### 3.4. Discovery of Key HCC Genes Based on Survival Analysis

**Figure 5:**
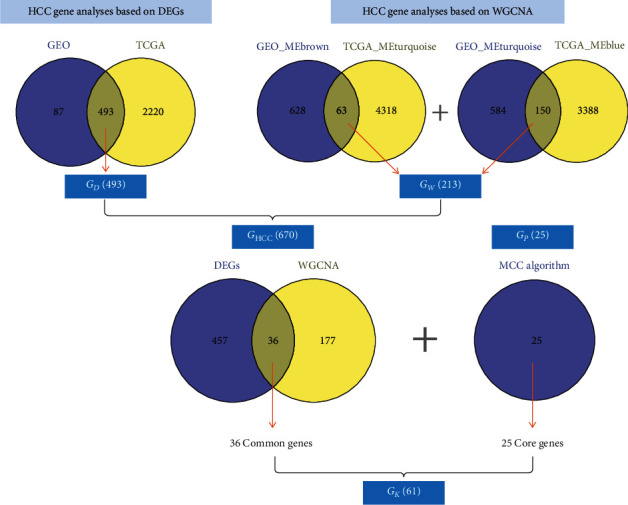
Flowchart of the key gene screening process.

**Figure 6:**
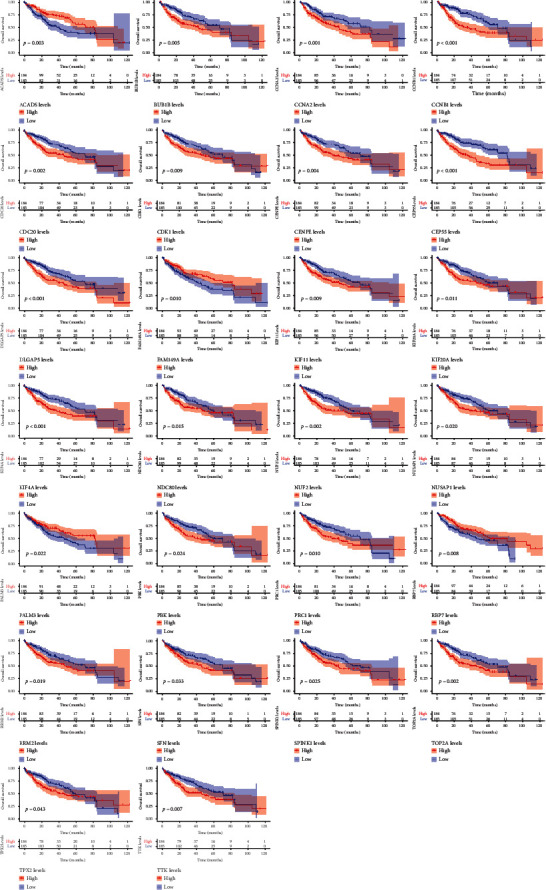
Survival curves of the 26 survival-related genes.

We obtained the intersection between the DEG set *G*_*D*_ and WGCNA gene set *G*_*W*_, obtaining 36 common genes. We then combined these genes with 25 core genes collected from the PPI network-based analyses, resulting in a total of 61 key genes (*G_k_*) on which we performed a survival analysis that revealed 26 genes related to survival in *G_k_* (Figure 5). These genes were ACADS, BUB1B, CCNA2, CCNB1, CDC20, CDK1, CENPE, CEP55, DLGAP5, FAM149A, KIF11, KIF20A, KIF4A, NDC80, NUF2, NUSAP1, PALM3, PBK, PRC1, RBP7, RRM2, SFN, SPINK1, TOP2A, TPX2, and TTK, as illustrated in Figure 6.

### 3.5. ADMET Assessment Results for Effective YCHD Ingredients

We conducted a literature analysis and find that of the 41 chemical components entered into blood, 5 components could not be retrieved from PubChem CID information. We derived data on 36 small YCHD molecules and subsequently performed ADMET assessments on the effective ingredients by using ACD/Labs and SwissADME. The corresponding structure of each small-molecule compound and its corresponding canonical SMILES were obtained from the PubChem database and then imported into the ACD/LABS software and the SwissADME online prediction system to evaluate these active ingredients.

In the assessments, we evaluated indicators such as gastrointestinal absorption, bioavailability (%), and dose (mg = 50), as presented in Table 1. Among the 36 molecules, 24 (66.7%) could be sufficiently absorbed by the stomach and intestines, and 22 (61.1%) had a bioavailability greater than 70%.

### 3.6. Results of the Prediction and Identification of Small YCHD Molecular Targets

We imported all small YCHD molecules into four online prediction systems (i.e., HitPick, SEA, SwissTargetPrediction, and STITCH) by using the method described in Section 2.8 to predict molecular targets. We then used thresholds to perform screening, obtaining 24 small YCHD molecules and 105 potential targets. Information on the targets is presented in Table 2.

**Figure 7:**
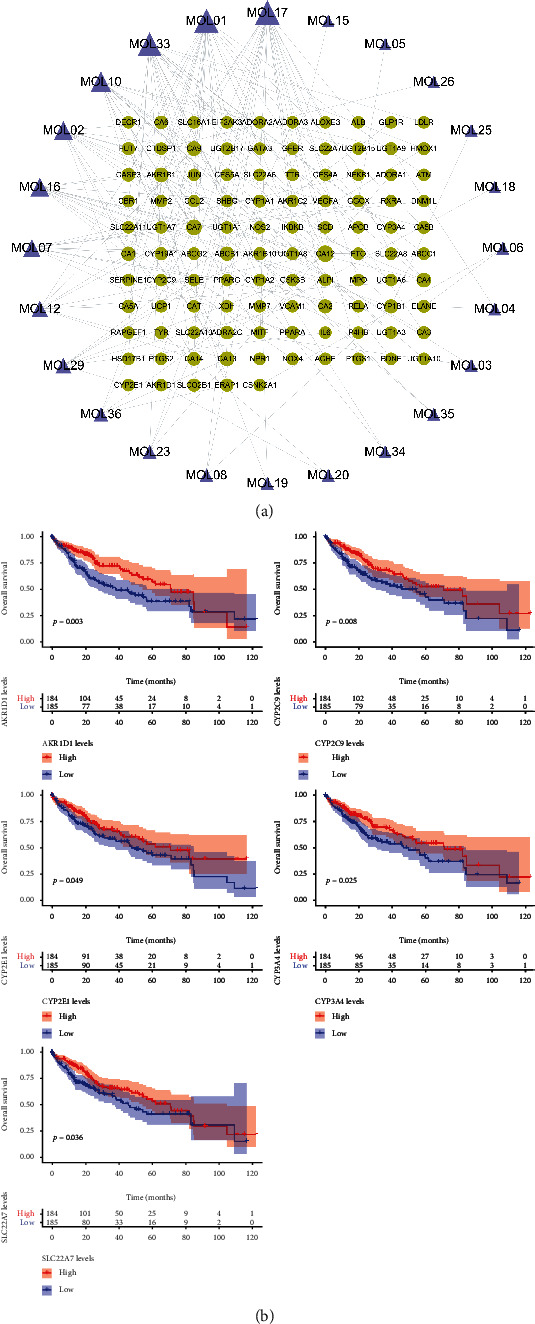
(a) Network of the 105 potential targets of 24 small YCHD molecules; the circles and triangles denote the targets and small molecules, respectively, and a greater node degree indicates a larger node size. (b) Five survival-related genes in YCHD targets.

We obtained the intersection between the 105 potential targets and the HCC disease gene set *G*_HCC_, which revealed that YCHD directly interfered with 17 HCC-related disease targets: AKR1B10, AKR1D1, CA2, CA5A, CCL2, CYP1A2, CYP2C9, CYP2E1, CYP3A4, PPARG, PTGS2, SELE, SERPINE1, SHBG, SLC22A7, UGT1A6, and XDH. A survival analysis performed on the 17 targets revealed that AKR1D1, CYP2C9, CYP2E1, CYP3A4, and SLC22A7 were related to the survival of patients with HCC (Figure 7(b)). The YCHD ingredient–target effect network is illustrated in Figure 7(a).

### 3.7. Network Analysis of YCHD Ingredients Interfering with HCC-Related Disease Targets

#### 3.7.1. Enrichment Analysis of YCHD Ingredients Interfering with HCC-Related Disease Targets

**Figure 8:**
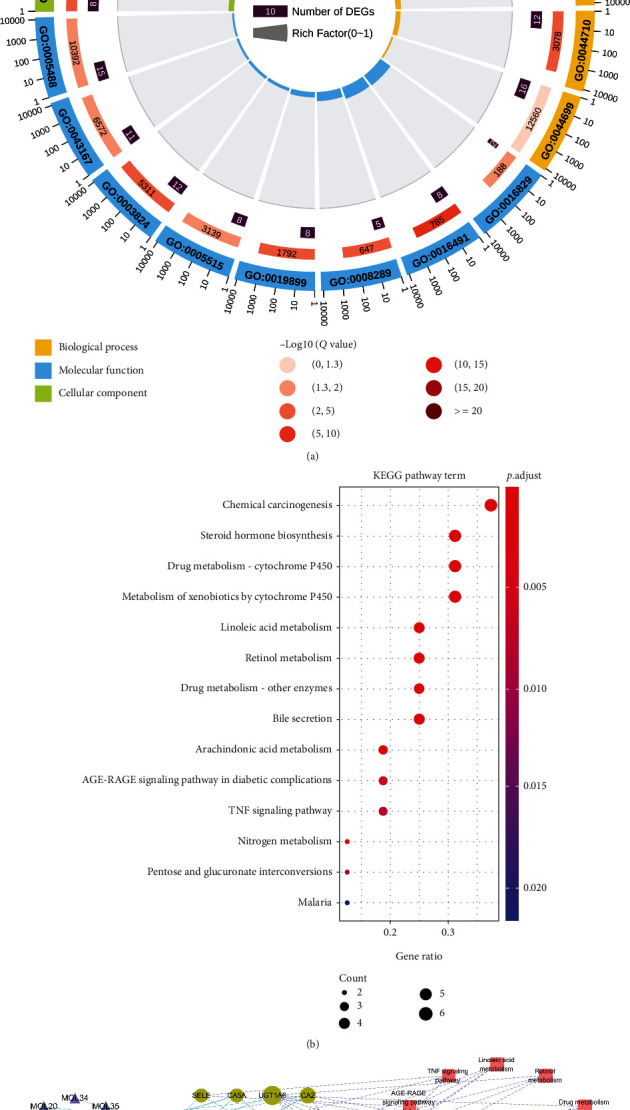
(a) Biological processes through which YCHD treats HCC. (b) KEGG pathways through which YCHD inhibits HCC. (c) YCHD ingredient–target–pathway network diagram. In (c), the triangles, circles, and squares represent small molecules, targets, and pathways, respectively, in which targets were enriched; the relationship between small molecules and targets and that between targets and pathways are represented by solid and dotted lines, respectively, and a greater node degree indicates a larger node size.

We performed an enrichment analysis on YCHD molecules and 17 HCC-related disease targets. A GO functional enrichment analysis revealed that the mechanisms underlying the effects of YCHD involved 17 biological processes, 7 molecular functions, and 9 cellular components (Figure 8(a)). The main YCHD effects included inhibition of abnormal cell proliferation, strengthening of the immune system, and facilitation of metabolism, possibly explaining the mechanism through which YCHD inhibits HCC. Through a KEGG enrichment analysis, we derived 14 pathways (Figure 8(b)): a cancer overview pathway (chemical carcinogenesis), an inflammatory signaling pathway (tumor necrosis factor (TNF) signaling pathway), and nine metabolic pathways (i.e., steroid hormone biosynthesis, drug metabolism—cytochrome P450, metabolism of xenobiotics by cytochrome P450, linoleic acid metabolism, retinol metabolism, drug metabolism—other enzymes, arachidonic acid metabolism, nitrogen metabolism, and pentose and glucuronate interconversions). Therefore, we deduced that the inflammatory signaling pathway and metabolic pathways were the most critical pathways for the delivery of the antiliver cancer effects of YCHD.

#### 3.7.2. Construction and Analysis of the Network of YCHD Ingredients Interfering with HCC-Related Disease Targets

To determine the targets and pathways regulated by YCHD compounds, we constructed a compound–target–pathway network incorporating all compounds, targets, and signaling pathways (Figure 8(c)).

As illustrated in Figure 8(c), salicylic acid (MOL17; degree = 5) exhibited effects on five targets, naringenin (MOL33; degree = 4) exhibited effects on four targets, and gallic acid (MOL01; degree = 3) exhibited effects on three targets; moreover, *UGT1A6* (degree = 9), *CYP3A4* (degree = 9), *CYP2E1* (degree = 8), *CYP2C9* (degree = 7), *CYP1A2* (degree = 7), and *CA2* (degree = 6) each contained one to two small molecules that exhibited effects on targets. However, many of these targets were enriched in multiple pathways. Of the 17 targets, 6 were enriched in the chemical carcinogenesis (degree = 6) signaling pathway.

The nitrogen metabolism and bile secretion signaling pathways (degree = 7) were linked to seven chemical molecules; chemical carcinogenesis (degree = 6) was linked to six chemical molecules; steroid hormone biosynthesis, drug metabolism—cytochrome P450, and xenobiotic metabolism by cytochrome P450 (degree = 5) were linked to five chemical molecules; retinol metabolism, drug metabolism—other enzymes, and linoleic acid metabolism (degree = 4) were linked to four chemical molecules; and the AGE-RAGE signaling pathway in diabetes, pentose and glucuronate interconversions, arachidonic acid metabolism, and the TNF signaling pathway (degree = 3) were linked to three chemical molecules. The remaining signaling pathways were linked to at least two chemical molecules.

### 3.8. Regulatory Network Analysis of YCHD Ingredients Interfering with Key Targets

*S*_*AB*_^*P*^ < 0 was used to determine whether small YCHD molecules affected and regulated the key target set in the *G_k_* set. The results indicated that 17 small YCHD molecules interfered with the key target set (Table 3).

**Figure 9:**
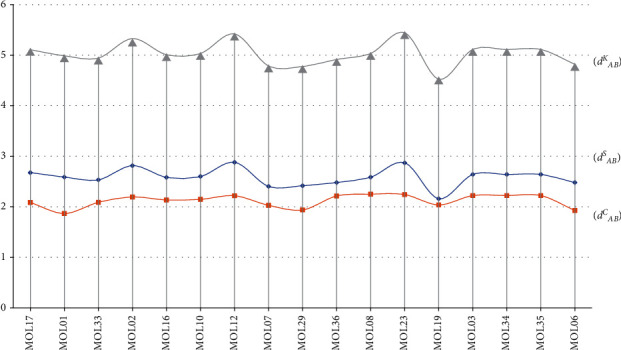
Comparison of the interference values of the 17 YCHD small molecules and those of the target set obtained using the three distance formulas.

The results obtained using three distance formulas revealed that the 17 small molecules had interference values similar to those of the key target set. Among the 17 small molecules, MOL02 (2,5-dimethyl-7-hydroxy chromone), MOL12 (7-methoxycoumarin), MOL17 (salicylic acid), and MOL23 (cirsimaritin) interfered with the target set most profoundly (Figure 9).

**Figure 10:**
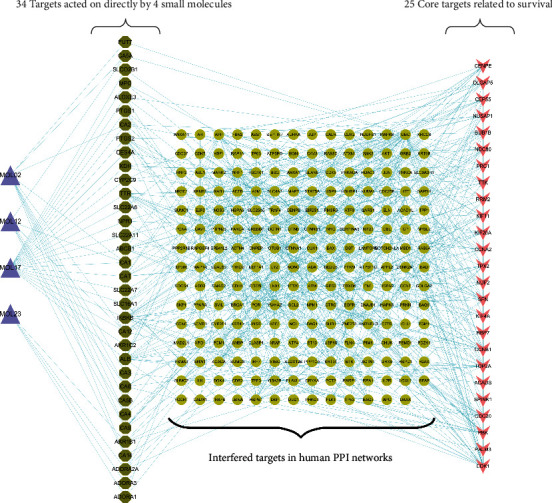
Targets acted on directly by four YCHD small molecules, core targets related to survival, and targets interfered with. Purple triangles represent the four small YCHD molecules, dark green octagons represent the 34 potential targets that the four small molecules directly acted on, ellipses represent the proteins that interfered with the 34 direct action targets in the human PPI network, and the V shapes represent the 25 key genes associated with HCC survival in *G_k_*.

Among the 34 targets directly acted on by the four small molecules, we discovered six genes, namely, *CA2*, *CA5A*, *CYP2C9*, *PTGS2*, *SLC22A7*, and *XDH*, to be related to HCC. In addition, *BCB1*, *ADORA1*, *ADORA2a*, *ADORA3*, *AKR1B1*, *AKR1C2*, *ALB*, *ALOXE3*, *CA1*, *CA12*, *CA14*, *CA3*, *CA4*, *CA5B*, *CA6*, *CA9*, *CAT*, *CES4A*, *FUT7*, *IKBKB*, *MPO*, *NPR1*, *PTGS1*, *SLC16A1*, *SLC22A11*, *SLC22A6*, *SLCO2B1*, and *TTR* were non-HCC-related genes (Figure 10). However, the results revealed the inherent complex mechanism of action, that is, these four small molecules could indirectly regulate 25 key genes related to HCC survival by disturbing 208 proteins in the PPI network by acting on these 34 targets.

## 4. Discussion

HCC is a complex disease whose onset and progression is related to multiple proteins and pathways [17]. TCM compounds contain numerous ingredients that affect multiple targets and exhibit diverse, multipathway pharmacological activities [18] that may assist in HCC treatment. Hanahan et al. proposed 10 theories of tumor hallmarks according to modern tumorigenesis and tumor development mechanisms [19]. Because TCM has a complex composition and affects various targets, it may simultaneously interfere with multiple tumor hallmarks.

TCM compounds contain numerous ingredients and involve complex mechanisms that may be linked to multiple targets and multiple pathways. In TCM, compounds without certain pharmacokinetic properties cannot reach the target organs; thus, they cannot effectively exhibit their biological activity. In a preliminary study, we observed that OB screening and DL assessments were the most favorable predictors of compounds with biological activity; however, the molecule effects were minimal if the molecule content was low or if the molecules metabolized quickly. In oral medication, ingredients that enter the bloodstream contribute the most to medicinal effects. Because of the complex characteristics of TCM, comprehensively studying the internal mechanisms of TCM compounds is difficult. Nevertheless, the use of bioinformatics methods to analyze TCM compound ingredients absorbed into the bloodstream may be a viable approach to determining the properties of such complex mechanisms. Thus, the current study employed such an approach to demonstrate the pharmacological mechanisms through which YCHD interferes with HCC.

YCHD is a traditional decoction commonly used in clinical practice. The current study used the network pharmacology method to study the anti-HCC effects of YCHD. We posit that YCHD demonstrates inhibitory effects on HCC by directly regulating the metabolism, inflammation, and signaling transduction pathways. Among the 36 YCHD ingredients absorbed into the bloodstream, 17 were directly related to liver cancer targets and inhibited HCC through interference with 14 pathways. For example, the inflammation-related signaling pathways have been identified to play a key role in the treatment and prevention of HCC [20]. Our research also revealed that MOL17 (salicylic acid), MOL29 (geniposide), and MOL33 (naringenin) can be used to treat HCC by regulating the TNF signaling pathway. Two studies have reported that geniposide [21] exerts anti-HCC effects by suppressing vascular endothelial growth factor expression and angiogenesis and that naringenin [22] suppresses the invasiveness and metastatic potential of HCC by inhibiting multiple signal transduction pathways. Energy metabolism may also play a critical role in the inhibition of HCC [23]. Our study revealed that 12 YCHD compounds affected 9 metabolism pathways. Five of these compounds can be used to treat HCC by regulating the energy metabolism pathway (i.e., MOL01 (gallic acid), MOL02 (2,5-dimethyl-7-hydroxychromone), MOL12 (7-methoxycoumarin), MOL16 (isoquercitrin), and MOL17 (salicylic acid)).

We identified 17 HCC targets directly affected by YCHD, of which 5 genes (AKR1D1, CYP2C9, CYP2E1, CYP3A4, and SLC22A7) were associated with the prognosis of patients with HCC. AKR1C3 plays crucial roles in multiple cancers, and a low expression level of AKR1D1 predicted poor prognosis and short median survival time [24]. CYP2C9 is involved in the metabolism of many carcinogens and drugs, and CYP2C9 was downregulated in human HCC progression [25]. CYP2E1 is a unique gene expressed in the liver but not expressed in HCC [26]. CYP3A4 expression is significantly low in the liver tumor tissue of patients with HCC [27]. Low SLC22A7 expression indicates a high risk of poor prognosis [28]. These five protein targets may be the prognostic targets for HCC and the targets of YCHD in the treatment of HCC. The network pharmacology analysis results demonstrate that MOL17 (salicylic acid) regulates SLC22A7 and CYP2C9, MOL20 (azelaic acid) regulates AKR1D1, MOL29 (geniposide) regulatesCYP2E1, and MOL33 (naringenin) regulates CYP3A4. In addition, gallic acid [29], physcion [30], rhein [31], isofraxidin [32], geniposide [21], naringenin [22], and chlorogenic acid [33] have been reported to exhibit anti-HCC effects.

The current study investigated how YCHD ingredients regulated and interfered with key targets; the results reveal similar trends in how the 17 small molecules interfered with the key target set. Among the 17 small molecules, MOL02 (2,5-dimethyl-7-hydroxychromone), MOL12 (7-methoxycoumarin), MOL17 (salicylic acid), and MOL23 (cirsimaritin) exhibited the most profound interference with the target set.

The current study adopted the network pharmacology method and used YCHD ingredients absorbed into the bloodstream to predict the targets of YCHD and construct meaningful pathways. In addition, the relationships between YCHD compounds, targets, and diseases were consolidated, and the multi-ingredient and multitarget characteristics of YCHD were analyzed. Finally, the mechanisms through which YCHD treat HCC were revealed.

In the current study, we combined bioinformatics and the network pharmacology method to predict which YCHD ingredients entered the bloodstream as well as the molecular targets and pathways through which YCHD treats HCC; the current results may serve as a basis for subsequent experimental studies. The specific mechanisms governing these ingredients should be examined through experiments in future studies.

## Figures and Tables

**Table 1:** ADMET assessment results and data on the 36 small YCHD molecules.

Number	Ingredient name	Molecular formula	PubChem CID	GI absorption	Bioavailability (%)
MOL01	Gallic acid	C7H6O5	370	High	7.73
MOL02	2,5-Dimethyl-7-hydroxy chromone	C11H10O3	5316891	High	99.36
MOL03	Cacticin	C22H22O12	5318644	Low	6.98
MOL04	Physcion	C16H12O5	10639	High	75.28
MOL05	Chimaphylin	C12H10O2	101211	High	89.68
MOL06	Chrysophanol	C15H10O4	10208	High	72.78
MOL07	Rhein	C15H8O6	10168	High	97.68
MOL08	Kaempferide	C16H12O6	5281666	High	98.78
MOL09	Villosolside	C16H26O9	127454	Low	3.69
MOL10	Scopoletin	C10H8O4	5280460	High	99.43
MOL11	4′-Hydroxyacetophenone	C8H8O2	7469	High	99.52
MOL12	7-Methoxycoumarin	C10H8O3	10748	High	99.57
MOL13	Safflor yellow A	C27H30O15	6448299	Low	0.02
MOL14	5,6-Dimethoxy-7-hydroxy coumarin	C11H10O5	5316862	High	99.5
MOL15	Isofraxidin	C11H15O5	5318565	High	99.41
MOL16	Isoquercitrin	C21H20O12	5280804	Low	2.58
MOL17	Salicylic acid	C7H6O3	338	High	83.24
MOL18	Isorhamnetin-3-glucoside	C22H22O12	14704554	Low	7.84
MOL19	Scoparone	C11H10O4	8417	High	99.61
MOL20	Azelaic acid	C9H16O4	2266	High	10.58
MOL21	6-Demethoxycapillarisin	C15H10O6	5316511	High	99.2
MOL22	Capillarisin	C16H12O7	5281342	High	97.83
MOL23	Cirsimaritin	C17H14O6	188323	High	99.37
MOL24	Capillartemisin	C19H24O4	6439717	High	98.77
MOL25	Rhamnocitrin	C16H12O6	5320946	High	99.2
MOL26	Gardenoside B	C17H24O11	24721095	Low	1.68
MOL27	Picrocrocinic acid	C16H26O8	5320582	Low	1.6
MOL28	Genipingentiobioside	C23H34O15	3082301	Low	0.35
MOL29	Geniposide	C17H24O10	107848	Low	5.72
MOL30	2,4-Decadienal	C10H16O	5283349	High	99.66
MOL31	2-Ethyl-2-hexenal	C8H14O	5354264	High	99.69
MOL32	Isosyringinoside	C23H34O14	131752947	Low	0.22
MOL33	Naringenin	C15H12O5	667495	High	98.38
MOL34	Neochlorogenic acid	C16H18O9	7067335	Low	0.48
MOL35	Chlorogenic acid	C16H18O9	1794427	Low	0.48
MOL36	6-Hydroxy-7-methoxy-2H-chromen-2-one	C10H8O4	69894	High	99.36

**Table 2:** YCHD molecular target information.

Numbers	Targets	Protein names	UniProt ID
Tar001	ABCB1	ATP-dependent translocase ABCB1	P08183
Tar002	ABCC1	Multidrug resistance-associated protein 1	P33527
Tar003	ABCG2	Broad substrate specificity ATP-binding cassette transporter ABCG2	Q9UNQ0
Tar004	ACHE	Acetylcholinesterase (AChE)	P22303
Tar005	ADORA1	Adenosine receptor A1	P30542
Tar006	ADORA2A	Adenosine receptor A2a	P29274
Tar007	ADORA3	Adenosine receptor A3	P0DMS8
Tar008	ADRA2C	Alpha-2C adrenergic receptor	P18825
Tar009	AKR1B1	Aldo-keto reductase family 1 member B1	P15121
Tar010	AKR1B10	Aldo-keto reductase family 1 member B10	O60218
Tar011	AKR1C2	Aldo-keto reductase family 1 member C2	P52895
Tar012	AKR1D1	Aldo-keto reductase family 1 member D1	P51857
Tar013	ALB	Albumin	P02768
Tar014	ALOXE3	Hydroperoxide isomerase ALOXE3	Q9BYJ1
Tar015	ALPI	Intestinal-type alkaline phosphatase	P09923
Tar016	APOB	Apolipoprotein B-100	P04114
Tar017	ATM	Serine-protein kinase ATM	Q13315
Tar018	BDNF	Brain-derived neurotrophic factor	P23560
Tar019	CA1	Carbonic anhydrase 1	P00915
Tar020	CA12	Carbonic anhydrase 12	O43570
Tar021	CA13	Carbonic anhydrase 13	Q8N1Q1
Tar022	CA14	Carbonic anhydrase 14	Q9ULX7
Tar023	CA2	Carbonic anhydrase 2	P00918
Tar024	CA3	Carbonic anhydrase 3	P07451
Tar025	CA4	Carbonic anhydrase 4	P22748
Tar026	CA5A	Carbonic anhydrase 5A	P35218
Tar027	CA5B	Carbonic anhydrase 5B	Q9Y2D0
Tar028	CA6	Carbonic anhydrase 6	P23280
Tar029	CA7	Carbonic anhydrase 7	P43166
Tar030	CA9	Carbonic anhydrase 9	Q16790
Tar031	CASP3	Caspase-3	P42574
Tar032	CAT	Catalase	P04040
Tar033	CBR1	Carbonyl reductase [NADPH] 1	P16152
Tar034	CCL2	C-C motif chemokine 2	P13500
Tar035	CES4A	Carboxylesterase 4A	Q5XG92
Tar036	CES5A	Carboxylesterase 5A	Q6NT32
Tar037	CSNK2A1	Casein kinase II subunit alpha	P68400
Tar038	CTDSP1	Carboxy-terminal domain RNA polymerase II polypeptide A small phosphatase 1	Q9GZU7
Tar039	CYP19A1	Aromatase	P11511
Tar040	CYP1A1	Cytochrome P450 1A1	P04798
Tar041	CYP1A2	Cytochrome P450 1A2	P05177
Tar042	CYP1B1	Cytochrome P450 1B1	Q16678
Tar043	CYP2C9	Cytochrome P450 2C9	P11712
Tar044	CYP2E1	Cytochrome P450 2E1	P05181
Tar045	CYP3A4	Cytochrome P450 3A4	P08684
Tar046	DECR1	2,4-Dienoyl-CoA reductase	Q16698
Tar047	DNM1L	Dynamin-1-like protein	O00429
Tar048	EIF2AK3	Eukaryotic translation initiation factor 2-alpha kinase 3	Q9NZJ5
Tar049	ELANE	Neutrophil elastase	P08246
Tar050	ERAP1	Endoplasmic reticulum aminopeptidase 1	Q9NZ08
Tar051	FTO	Alpha-ketoglutarate-dependent dioxygenase FTO	Q9C0B1
Tar052	FUT7	Alpha-(1,3)-fucosyltransferase 7	Q11130
Tar053	GATA3	Trans-acting T-cell-specific transcription factor GATA-3	P23771
Tar054	GFER	FAD-linked sulfhydryl oxidase ALR	P55789
Tar055	GGCX	Vitamin K-dependent gamma-carboxylase	P38435
Tar056	GLP1R	Glucagon-like peptide 1 receptor	P43220
Tar057	GSK3B	Glycogen synthase kinase-3 beta	P49841
Tar058	HMOX1	Heme oxygenase 1	P09601
Tar059	HSD17B1	Estradiol 17-beta-dehydrogenase 1	P14061
Tar060	IKBKB	Inhibitor of nuclear factor kappa-B kinase subunit beta	O14920
Tar061	IL6	Interleukin-6	P05231
Tar062	JUN	Transcription factor AP-1	P05412
Tar063	LDLR	Low-density lipoprotein receptor	P01130
Tar064	MITF	Microphthalmia-associated transcription factor	O75030
Tar065	MMP2	Matrix metalloproteinase-2	P08253
Tar066	MMP7	Matrix metalloproteinase-7	P09237
Tar067	MPO	Myeloperoxidase	P05164
Tar068	NFKB1	Nuclear factor NF-kappa-B p105 subunit	P19838
Tar069	NOS2	Nitric oxide synthase	P35228
Tar070	NOX4	NADPH oxidase 4	Q9NPH5
Tar071	NPR1	Atrial natriuretic peptide receptor 1	P16066
Tar072	P4HB	Protein disulfide-isomerase	P07237
Tar073	PPARA	Peroxisome proliferator-activated receptor alpha	Q07869
Tar074	PPARG	Peroxisome proliferator-activated receptor gamma	P37231
Tar075	PTGS1	Prostaglandin G/H synthase 1	P23219
Tar076	PTGS2	Prostaglandin G/H synthase 2	P35354
Tar077	RAPGEF1	Rap guanine nucleotide exchange factor 1	Q13905
Tar078	RELA	Transcription factor p65	Q04206
Tar079	RXRA	Retinoic acid receptor RXR-alpha	P19793
Tar080	SCD	Acyl-CoA desaturase	O00767
Tar081	SELE	E-selectin	P16581
Tar082	SERPINE1	Plasminogen activator inhibitor 1	P05121
Tar083	SHBG	Sex hormone-binding globulin	P04278
Tar084	SLC16A1	Monocarboxylate transporter 1	P53985
Tar085	SLC22A10	Solute carrier family 22 member 10	Q63ZE4
Tar086	SLC22A11	Solute carrier family 22 member 11	Q9NSA0
Tar087	SLC22A6	Solute carrier family 22 member 6	Q4U2R8
Tar088	SLC22A7	Solute carrier family 22 member 7	Q9Y694
Tar089	SLC22A8	Solute carrier family 22 member 8	Q8TCC7
Tar090	SLCO2B1	Solute carrier organic anion transporter family member 2B1	O94956
Tar091	TTR	Transthyretin	P02766
Tar092	TYR	Tyrosinase	P14679
Tar093	UCP1	Mitochondrial brown fat uncoupling protein 1	P25874
Tar094	UGT1A1	UDP-glucuronosyltransferase 1A1	P22309
Tar095	UGT1A10	UDP-glucuronosyltransferase 1A10	Q9HAW8
Tar096	UGT1A3	UDP-glucuronosyltransferase 1A3	P35503
Tar097	UGT1A6	UDP-glucuronosyltransferase 1-6	Q64435
Tar098	UGT1A7	UDP-glucuronosyltransferase 1A7	Q9HAW7
Tar099	UGT1A8	UDP-glucuronosyltransferase 1A8	Q9HAW9
Tar100	UGT1A9	UDP-glucuronosyltransferase 1A9	Q62452
Tar101	UGT2B15	UDP-glucuronosyltransferase 2B15	P54855
Tar102	UGT2B17	UDP-glucuronosyltransferase 2B17	O75795
Tar103	VCAM1	Vascular cell adhesion protein 1	P19320
Tar104	VEGFA	Vascular endothelial growth factor A	P15692
Tar105	XDH	Xanthine dehydrogenase/oxidase	P47989

**Table 3:** Regulatory network analyses and calculation results of small YCHD molecules interfering with key targets.

MOL	*d*_*AB*_^*S*^	*d*_*AB*_^*C*^	*d*_*AB*_^*K*^	*S*_*AB*_^*P*^
MOL17	2.68	2.09	5.11	**-2.09**
MOL01	2.59	1.87	4.99	**-2.01**
MOL33	2.53	2.09	4.94	**-1.98**
MOL02	**2.81**	2.19	5.33	**-1.95**
MOL16	2.58	2.14	5.01	**-1.84**
MOL10	2.60	2.15	5.03	**-1.75**
MOL12	**2.88**	**2.22**	**5.42**	**-1.71**
MOL07	2.40	2.03	4.79	**-1.54**
MOL29	2.42	1.94	4.78	**-1.53**
MOL36	2.48	2.21	4.91	**-0.98**
MOL08	2.59	2.25	5.03	**-0.88**
MOL23	**2.87**	2.24	5.44	**-0.86**
MOL19	2.16	2.04	4.52	**-0.64**
MOL03	2.64	2.22	5.11	**-0.49**
MOL34	2.64	2.22	5.11	**-0.49**
MOL35	2.64	2.22	5.11	**-0.49**
MOL06	2.48	1.93	4.82	**-0.32**

## Data Availability

The data used to support the findings of this study are included within the article.

## References

[1] Chen W., Zheng R., Baade P. D. (2016). Cancer statistics in China, 2015. *CA: a Cancer Journal for Clinicians*.

[2] Daher S., Massarwa M., Benson A. A., Khoury T. (2018). Current and future treatment of hepatocellular carcinoma: an updated comprehensive review. *Journal of Clinical and Translational Hepatology*.

[3] Hu Y., Wang S., Wu X. (2013). Chinese herbal medicine-derived compounds for cancer therapy: a focus on hepatocellular carcinoma. *Journal of Ethnopharmacology*.

[4] Huang J., Cheung F., Tan H. Y. (2017). Identification of the active compounds and significant pathways of yinchenhao decoction based on network pharmacology. *Molecular Medicine Reports*.

[5] Xu X., Zhang W., Huang C. (2012). A novel chemometric method for the prediction of human oral bioavailability. *International Journal of Molecular Sciences*.

[6] Tao W., Xu X., Wang X. (2013). Network pharmacology-based prediction of the active ingredients and potential targets of Chinese herbal *Radix Curcumae* formula for application to cardiovascular disease. *Journal of Ethnopharmacology*.

[7] Sun H., Zhang A.-h., Le Yang M.-x. L., Fang H., Xie J., Wang X.-j. (2019). High-throughput chinmedomics strategy for discovering the quality-markers and potential targets for Yinchenhao decoction. *Phytomedicine*.

[8] Wu D., Chen X., Hu S., Bai X. (2017). Study on major antitumor components in Yinchenhao decoction in vitro and in vivo based on hollow fiber cell fishing coupled with high performance liquid chromatography. *Journal of Chromatography B*.

[9] Ma Y.-m., Zhang X.-z., Su Z.-z. (2015). Insight into the molecular mechanism of a herbal injection by integrating network pharmacology and in vitro. *Journal of Ethnopharmacology*.

[10] Boran A. D., Iyengar R. (2010). Systems pharmacology. *Mount Sinai Journal of Medicine: A Journal of Translational and Personalized Medicine*.

[11] Luo T.-t., Lu Y., Yan S.-k., Xiao X., Rong X.-l., Guo J. (2020). Network pharmacology in research of Chinese medicine formula: methodology, application and prospective. *Chinese Journal of Integrative Medicine*.

[12] Guo W., Huang J., Wang N. (2019). Integrating network pharmacology and pharmacological evaluation for deciphering the action mechanism of herbal formula zuojin pill in suppressing hepatocellular carcinoma. *Frontiers in Pharmacology*.

[13] Huang J., Guo W., Cheung F., Tan H. Y., Wang N., Feng Y. (2020). Integrating network pharmacology and experimental models to investigate the efficacy of coptidis and scutellaria containing huanglian jiedu decoction on hepatocellular carcinoma. *The American Journal of Chinese Medicine*.

[14] Huang J., Chen F., Zhong Z. (2020). Interpreting the pharmacological mechanisms of huachansu capsules on hepatocellular carcinoma through combining network pharmacology and experimental evaluation. *Frontiers in Pharmacology*.

[15] Clough E., Barrett T. (2016). The Gene Expression Omnibus database. *Molecular Biology*.

[16] Tomczak K., Czerwińska P., Wiznerowicz M. (2015). Review the Cancer Genome Atlas (TCGA): an immeasurable source of knowledge. *Współczesna Onkologia*.

[17] Feitelson M. A., Sun B., Tufan N. L. S., Liu J., Pan J., Lian Z. (2002). Genetic mechanisms of hepatocarcinogenesis. *Oncogene*.

[18] Zhang W., Huai Y., Miao Z., Qian A., Wang Y. (2019). Systems pharmacology for investigation of the mechanisms of action of traditional Chinese medicine in drug discovery. *Frontiers in Pharmacology*.

[19] Hanahan D., Weinberg R. . A. (2011). Hallmarks of cancer: the next generation. *Cell*.

[20] Wang J., Tokoro T., Higa S., Kitajima I. (2006). Anti-inflammatory effect of pitavastatin on NF-kappa B activated by TNF-alpha in hepatocellular carcinoma cells. *Biological and Pharmaceutical Bulletin*.

[21] Zhang C., Wang N., Tan H.‐. Y. (2020). Direct inhibition of the TLR4/MyD88 pathway by geniposide suppresses HIF-1*α*-independent VEGF expression and angiogenesis in hepatocellular carcinoma. *British Journal of Pharmacology*.

[22] Yen H. R., Liu C. J., Yeh C. C. (2015). Naringenin suppresses TPA-induced tumor invasion by suppressing multiple signal transduction pathways in human hepatocellular carcinoma cells. *Chemico-Biological Interactions*.

[23] Chen Q., Li F., Gao Y., Xu G., Liang L., Xu J. (2020). Identification of energy metabolism genes for the prediction of survival in hepatocellular carcinoma. *Frontiers in Oncology*.

[24] Zhu P., Feng R., Lu X. (2021). Diagnostic and prognostic values of AKR1C3 and AKR1D1 in hepatocellular carcinoma. *Aging*.

[25] Yu D., Green B., Marrone A. (2015). Suppression of CYP2C9 by micro RNA hsa-mi R-128-3p in human liver cells and association with hepatocellular carcinoma. *Scientific Reports*.

[26] Man X. B., Tang L., Qiu X. H. (2004). Expression of cytochrome P4502E1 gene in hepatocellular carcinoma. *World Journal of Gastroenterology*.

[27] Flannery P. C., Abbott K. L., Pondugula S. R. (2020). Correlation of PPM1A downregulation with CYP3A4 repression in the tumor liver tissue of hepatocellular carcinoma patients. *European Journal of Drug Metabolism and Pharmacokinetics*.

[28] Yasui Y., Kudo A., Kurosaki M. (2014). Reduced organic anion transporter expression is a risk factor for hepatocellular carcinoma in chronic hepatitis C patients: a propensity score matching study. *Oncology*.

[29] Sun G., Zhang S., Xie Y., Zhang Z., Zhao W. (2016). Gallic acid as a selective anticancer agent that induces apoptosis in SMMC-7721 human hepatocellular carcinoma cells. *Oncology Letters*.

[30] Pan X.-p., Wang C., Li Y., Huang L.-h. (2018). Physcion induces apoptosis through triggering endoplasmic reticulum stress in hepatocellular carcinoma. *Biomedicine & Pharmacotherapy*.

[31] Shi P., Huang Z., Chen G. (2008). Rhein induces apoptosis and cell cycle arrest in human hepatocellular carcinoma BEL-7402 Cells. *The American Journal of Chinese Medicine*.

[32] Yamazaki T., Tokiwa T. (2010). Isofraxidin, a coumarin component from Acanthopanax senticosus, inhibits matrix metalloproteinase-7 expression and cell invasion of human hepatoma cells. *Biological and Pharmaceutical Bulletin*.

[33] Yan Y., Liu N., Hou N., Dong L., Li J. (2017). Chlorogenic acid inhibits hepatocellular carcinoma in vitro and in vivo. *The Journal of Nutritional Biochemistry*.

